# Perceived Benefits and Barriers for Autistic Adults Accessing Therapeutic Horse Riding for Mental Health

**DOI:** 10.3390/bs16010084

**Published:** 2026-01-07

**Authors:** Hannah Louise Brumpton, Niko Kargas

**Affiliations:** 1 School of Psychology Sport Science & Wellbeing, College of Health and Science, University of Lincoln, Lincoln LN6 7TS, UK; hannah.brumpton@hotmail.co.uk; 2 Department of Cognitive Sciences, College of Humanities and Social Sciences, United Arab Emirates University, Al Ain P.O. Box 15551, United Arab Emirates

**Keywords:** autism, autistic adults, therapeutic horse riding, mental well-being, access barriers, embodied engagement, relational safety

## Abstract

Therapeutic horse riding (THR) is a non-traditional intervention that may support mental well-being in individuals with autism spectrum conditions. Despite growing interest, most research has focused on children and has tended to privilege practitioner or caregiver perspectives, leaving autistic adults underrepresented. This qualitative study explores the psychological benefits and systemic barriers associated with THR among Autistic adults, drawing on perspectives from both clients and practitioners. Semi-structured interviews were conducted with six Autistic clients and four practitioners, and the data were analysed using reflexive thematic analysis. Five overarching themes were constructed: Facing the Puissance: barriers to accessing THR, Pathways to Participation, Embodied Engagement, To Understand and To Be Understood, and Beyond the Arena—Impacts That Last. Participants described enjoyment, increased confidence, and a sense of achievement, with effects accumulating over time and often extending beyond the riding arena into daily life. Barriers included cost, accessibility, and limited availability of appropriately trained staff and facilities. These findings add to the limited evidence base on THR for Autistic adults by providing an in-depth, contextually grounded account of participants’ experiences. They suggest that, for verbally fluent Autistic adults who choose to access THR in similar settings, THR can enhance well-being, self-agency, and relationship-building, whilst also revealing structural obstacles that restrict equitable access.

## 1. Introduction

Equine-assisted services (EAS) is increasingly used as an umbrella term for structured human–horse programmes, encompassing therapeutic riding, equine-assisted learning, equine-assisted psychotherapy, and related activities. Recent American consensus work has recommended adopting EAS as a field-level descriptor, with more specific terms used for distinct service models (e.g., equine-assisted psychotherapy, therapeutic riding). In line with this terminology, we use EAS to refer to the broader field and therapeutic horse riding (THR) to describe mounted, psychologically focused sessions in which the horse–human relationship is used intentionally to support mental well-being rather than physical rehabilitation (Wood et al., 2021). Positioning THR within this framework facilitates comparison with emerging EAS research internationally and clarifies that the present study focuses on a mental-health-oriented, rather than purely recreational or sport-focused, form of riding. While EAS have gained increasing recognition, existing research has predominantly focused on children and young people, often emphasizing the perspectives of caregivers or practitioners rather than those of Autistic individuals themselves (Malcolm et al., 2018). Previous studies have highlighted the need for research that directly incorporates the voices of Autistic people (Botha & Cage, 2022; Holt et al., 2022; McDaniel Peters & Wood, 2017; Saunders, 2018; Silberman, 2017). Moreover, the limited qualitative literature in EAS that does engage Autistic participants has largely concentrated on young females, with minimal attention given to adults (Warner et al., 2022). Accordingly, the present study aimed to explore the perceived benefits and barriers encountered by Autistic adults in accessing therapeutic horse riding (THR) as a means of supporting mental well-being. To address this gap in the literature, the study foregrounds first-person narratives from Autistic adults, complemented by professional insights from THR practitioners. By integrating these perspectives, the research offers a more comprehensive understanding of THR’s potential to enhance psychological well-being, while also highlighting the relational and structural challenges that may affect access. This approach situates THR within broader discourses on neurodiversity, accessibility, and the provision of mental health services for Autistic adults, a population that remains underrepresented in mainstream research, healthcare provisions and relevant support systems (Jones et al., 2025 Kargas et al., 2023; Moore et al., 2024; UK Parliament, 2023).

Autism Spectrum Disorder (ASD) is characterised in diagnostic systems such as the ICD-11 (World Health Organization, 2022) and DSM-5-TR (APA, 2025) by social demands that exceed the individual’s adaptive capacities and by restricted and repetitive patterns of behaviour. There are an estimated 700,000 Autistic people in the UK (Buescher et al., 2014; Centres for Disease Control and Prevention, 2022; National Autistic Society, 2024), with higher estimates when including those without formal diagnosis. Autistic adults face enduring disadvantages compared to typically developing peers (Howlin & Magiati, 2017), including lower self-esteem (Nguyen et al., 2020), reduced confidence, particularly in those diagnosed in adulthood (Corden et al., 2021; Darazsdi & Bialka, 2023; Milton & Sims, 2016), and wider economic and social barriers (Fletcher-Watson, 2024; Rogge & Janssen, 2019; Seltzer et al., 2003; Zhao et al., 2023). Horses have been domesticated for much of recorded history, and in recent years, they have increasingly been recognised as valuable partners in therapeutic interventions aimed at supporting mental health and well-being (Anastasya et al., 2024; Cleary et al., 2024; Hatcher et al., 2019; Ward et al., 2022; Xiao et al., 2023). Equine-assisted services are expanding, with approximately 280 individuals currently registered with the Athena practitioner register (Athena, 2025), and nearly 200 businesses and associates listed on the Human Equine Interaction Register (HETI, 2025). However, many practitioners operate outside these formal registries, reflecting the growing scope and diversification of the field (Seery & Wells, 2024).

The therapeutic potential of human–animal interactions has long been acknowledged, with documented inclusion of animals in treatment settings dating back to 1792 (McCulloch, 1983; Morrison, 2007). Empirical research has demonstrated that interactions with horses can enhance self-confidence, self-esteem, and overall psychological well-being (Bizub et al., 2003; Burgon, 2011; Cantin & Marshall-Lucette, 2011; Karol, 2007; Hatcher et al., 2019). For instance, participants often report initial apprehension when engaging with horses, which can evolve into increased self-efficacy and a sense of empowerment through participation in equine-assisted programmes (Bizub et al., 2003). Feelings of calmness, enjoyment and confidence have also been reported by autistic children when riding horses (Browne et al., 2025). Within autism research, equine-assisted programmes have most commonly been studied with children and adolescents. Quantitative and mixed-method studies of therapeutic riding and related equine-assisted activities report improvements in social communication, adaptive behaviour, and motor skills, alongside reductions in anxiety and challenging behaviours (e.g., Gabriels et al., 2015; Xiao et al., 2023). Qualitative and mixed-method work with autistic children similarly highlights increased enjoyment, confidence, and emotion regulation, as well as the perceived kindness and safety offered by practitioners and horses (Burgon, 2011; Malcolm et al., 2018; Browne et al., 2025; Cleary et al., 2024). However, autistic adults’ own accounts of engaging with THR remain sparse, and adult-focused studies have tended to privilege practitioner or caregiver perspectives (McDaniel Peters & Wood, 2017; Warner et al., 2022). There is therefore limited understanding of how Autistic adults themselves perceive the benefits, challenges, and broader life impacts of THR.

Despite the promising outcomes associated with equine-assisted interventions, research in this area faces several methodological and conceptual challenges. Studies often rely on small sample sizes, yield inconsistent findings, and employ varied definitions and delivery models of equine-assisted services (Bachi, 2012; Lentini & Knox, 2009). Moreover, much of the existing literature focuses on structured, therapy-oriented sessions, leaving the potential benefits of broader, informal horse–human interactions underexplored (Burgon, 2011; Cantin & Marshall-Lucette, 2011; Karol, 2007). Access to therapeutic horse riding is further constrained by financial and logistical barriers. Sessions can be prohibitively expensive (Burgon, 2011; Kendall et al., 2014), and funding opportunities remain limited (Kendall et al., 2014). These challenges are particularly salient for Autistic adults, who are disproportionately affected by unemployment and economic disadvantage (Farkas et al., 2021; Harmuth et al., 2018; NICE, 2025). As such, it is essential to investigate both the benefits and barriers associated with therapeutic horse riding (THR) for this population, in order to inform equitable service provision and support evidence-based funding decisions.

In this context, the current study focuses specifically on THR, conceptualised as a mental-health-oriented equine-assisted service, delivered by practitioners with qualifications in mental health and extensive equestrian experience. We explore the perceived benefits and barriers of THR among Autistic adults who have chosen to access a THR programme at a single UK centre, alongside the perspectives of practitioners delivering the service. The Autistic participants were all verbally fluent and able to provide informed consent, reflecting a subgroup of adults who are already engaged with support. Rather than evaluating the efficacy of THR or seeking statistically generalisable findings, the study offers an in-depth, contextually grounded account of how THR is experienced and understood by these Autistic adults and the practitioners who work with them.

## 2. Materials and Methods

### 2.1. Design

The study employed a qualitative design using semi-structured interviews to enable participants to reflect on and elaborate upon their experiences of THR for mental health (Kallio et al., 2016). The flexible format and adaptable sequencing of questions facilitated in-depth discussions and the generation of rich, nuanced data, while allowing participants autonomy over the extent to which they engaged with specific topics, thereby respecting individual comfort levels. Furthermore, the semi-structured format enabled researchers to tailor the phrasing and delivery of questions to accommodate participants’ communication preferences and potential verbal processing needs, thereby enhancing the accessibility and inclusivity of the interview process.

### 2.2. Setting and THR Programme

Data were collected at a single UK THR centre that offers equine-assisted services for adults and young people with mental health difficulties and/or neurodivergent conditions. The service is delivered within a small, not-for-profit setting that combines mental-health training and extensive equestrian experience. THR sessions are explicitly framed as mental-health-oriented rather than sport- or rehabilitation-focused, and are delivered by practitioners with relevant professional backgrounds (e.g., counselling, psychotherapy, psychology, and equine-assisted practice). A typical THR session at the centre involves meeting the horse and practitioner, grooming and preparing the horse, mounted work in an arena, and a period of reflection and discussion. Sessions are tailored to the needs and goals of each client, with attention paid to sensory preferences, communication styles, and emotional safety. Clients usually attend on a regular basis, although frequency varies (e.g., weekly, fortnightly, or monthly) depending on personal circumstances and funding. All Autistic clients and practitioners in this study were recruited from this single centre, and the implications of this single-site design for transferability are considered in the Discussion.

### 2.3. Researcher Position

The primary researcher for this study was a white British female enrolled in the MSc Developmental Psychology programme at the University of Lincoln. As Braun and Clarke (2013) note, researchers may occupy both insider and outsider positions within the research context. In this study, the researcher held a dual positionality. Her involvement as a therapeutic riding practitioner at the THR facility attended by some participants positioned her as an insider with detailed contextual knowledge and pre-existing relationships with several clients and all practitioners. Conversely, as someone without a formal Autism diagnosis and who does not self-identify as Autistic, she also occupied an outsider position in relation to participants’ lived experiences of autism. In the context of practitioner interviews, her primary role as a therapeutic riding practitioner further reinforced her insider status.

This dual role has potential advantages and challenges. The existing trust and familiarity may have facilitated open conversation, but may also have made participants more likely to emphasise positive aspects of THR or to downplay criticisms, particularly among practitioners whose employment is tied to the programme. To address these issues, the researcher engaged in ongoing reflexive practice (see Section 2.6), explicitly considered how her practitioner role, values, and expectations might shape the research process, and used supervision meetings to critically examine emerging assumptions and interpretations. During interviews, she actively invited participants to discuss challenges, barriers, and negative or ambivalent experiences, and emphasised that participation was voluntary, confidential, and independent of their access to services.

### 2.4. Ethics and Recruitment

Ethical approval was granted by the University of Lincoln (Approval No. UoL2025_19424), and the study was conducted in accordance with the Declaration of Helsinki. All participants were recruited via a gatekeeper at the THR centre to ensure ethical recruitment and to protect clients from feeling pressured to participate. Theoretical sampling was used to ensure that participants’ experiences were relevant to the research questions, focusing on Autistic adults who had accessed THR for mental health and practitioners who delivered such sessions. Seven Autistic clients expressed interest and six consented to be interviewed. Five practitioners expressed interest and four consented. No financial incentives were offered. Accessibility and participant welfare were prioritised before, during, and after the interviews. Participants were informed that they could pause, stop, or resume the interview at any time, decline to answer any questions, or bring another person for support if desired. All participants provided written informed consent and were reminded that they could withdraw their data up to seven days after their interview, at which point transcripts were finalised and anonymised. At the end of each interview with Autistic adults, the researcher checked that the participant felt comfortable leaving and provided a debrief sheet with mental-health signposting. All participants were recruited from the same THR centre described in Section 2.2. This single-site recruitment enhances contextual specificity but limits variation in service models and organisational cultures; the implications for transferability are discussed later.

### 2.5. Participants

#### 2.5.1. Autistic Adults

Six Autistic adult women who had accessed THR participated in the study. In line with the inclusive, neurodiversity-affirming ethos of the project, participants were eligible if they either had a formal diagnosis of autism spectrum condition (*n* = 5) or self-identified as Autistic (*n* = 1); both groups are therefore included under the term “Autistic adults” in this paper. All were aged over 18 years (range 25–57 years; Mdn = 30, IQR = 12). All Autistic participants were able to provide written informed consent and engage in a semi-structured interview, indicating average or above-average intellectual and verbal functioning. However, no formal psychometric or clinical data were collected to specify participants’ cognitive profiles or to categorize them within traditional diagnostic frameworks. Consequently, the findings primarily reflect the perspectives of Autistic adults with relatively strong verbal and cognitive skills who were already engaged with a specialist THR service. As discussed later, this limits the extent to which the results can be transferred to Autistic individuals with higher support needs or significant communication challenges.

Eligibility criteria required participants to be over 18 years of age and to have attended a minimum of three THR sessions within the previous twelve months. In addition, four THR practitioners who had experience delivering sessions to Autistic adults were interviewed.

#### 2.5.2. Practitioners

Four female THR practitioners who had experience delivering sessions to Autistic adults were also interviewed. Practitioner participants were required to be over 18 years of age and to have provided THR services to Autistic adults at the centre. Their experience in THR with Autistic adults ranged from approximately three months to five years. Practitioner participants were also required to be over the age of 18 and to have provided THR services to Autistic adults; their experience ranged from three months to five years. Practitioners came from a range of professional backgrounds (e.g., counselling, psychology, equine-assisted services) and were actively engaged in efforts to develop a more evidence-based approach to THR. Their investment in the programme and interest in generating an evidence base may have contributed to a generally positive orientation towards THR in their accounts; this potential bias is considered in the Reflexivity, Analysis, and Discussion sections. The researcher aimed to achieve data saturation while acknowledging the potential limitations imposed by the small sample size, which reflects the broader challenges associated with participant recruitment in this field.

### 2.6. Interview Guide: Development and Content

The semi-structured interview protocol was developed collaboratively with input from multiple stakeholder groups. The Principal Investigator drafted the initial topic guide based on the existing literature on equine-assisted interventions and autism, the aims of the study, and clinical experience in THR. This draft guide was then reviewed by colleagues at the Autism Research Innovation Centre (ARIC) at the University of Lincoln, by members of the Progressive Autism Group, a user-led group comprising Autistic university students and by experienced THR practitioners at the centre. Their feedback focused on ensuring that questions were clear, non-patronising, and respectful of Autistic perspectives, avoiding deficit-based language, and to ensure that the questions were meaningful and acceptable from a service-delivery perspective. Together, these iterative consultations led to refinements in wording, ordering, and emphasis, and helped ensure that the guide was both clinically relevant and aligned with Autistic priorities.

The same core semi-structured guide was used across both participant groups to support consistency, with adaptations in emphasis and follow-up prompts to reflect participants’ roles (Autistic client or practitioner). Initial questions with Autistic clients aimed to ease into the interview with simple questions such as “Can you start by telling me how you first learnt about and accessed THR for mental health?” and from there building a rapport with the participant. Further questions such as “Are there any short-term impacts, either positive or negative from attending THR?” were based on previous literature findings which found a range of both positive and negative emotions may be felt (Burgon, 2011; Hatcher et al., 2019; Karol, 2007). For practitioners, parallel questions explored their role, experiences working with Autistic adults in THR, perceived benefits and harms, and systemic and practical barriers to access, including their views on the evidence base and what might be needed to strengthen it. In line with best practice in autism research, the interview questions were shared with all participants in advance (e.g., via email or as part of the information pack). This was intended to reduce anxiety, allow time for processing and preparation, and support participants in deciding what they did and did not wish to discuss. To enhance transparency, the full set of interview questions is provided as Supplementary Materials.

### 2.7. Interview Procedure

All interviews were conducted in person in private rooms at the stables, chosen to maximise participant comfort by situating conversations in a familiar, low-demand environment close to where THR sessions typically take place. Interviews were scheduled at times that suited participants and, where possible, avoided busy yard periods to minimise sensory overload. We recognise that conducting interviews on-site may have influenced responses (for example, by reinforcing participants’ identification with the centre or making them less likely to express criticism), and we consider this in interpreting the findings.

Interviews were audio-recorded via Microsoft Teams on a secure laptop. Automated transcripts were generated by the platform and subsequently reviewed and manually corrected by the researcher to ensure accuracy. Interviews were completed on different days to avoid interviewer fatigue and to allow time for reflexive noting between sessions. Interview lengths ranged from 19 min 7 s to 44 min 43 s.

Prior to the interview, the participants were given a participant consent form to read and sign. This was an opportunity to ask the researcher any questions. The consent form contained information on withdrawal, which could be requested up to 7 days after the completed interview. Following this window, transcripts were anonymised by removing identifying information such as names and locations. Participants were informed that they were not required to answer any questions they did not want to, and they could go into as much or as little detail as they desired. Participants were also invited to raise any additional issues they considered important. The current study also explored longer-term effects of THR on the well-being of Autistic adults. Practitioners received the same consent form and participant information sheet; however their questions differed. Firstly, asking them to outline their role and experiences working with Autistic adults in the context of THR, to allow the interviewer to understand their role. The interview then moved on to what barriers the practitioners felt were preventing, or making access to THR harder for Autistic adults, before discussing ways of working with or removing these barriers where possible. Practitioners were also asked to expand on short- and long-term impacts of THR, and finally given the opportunity to share any thoughts they felt were valuable.

### 2.8. Reflexivity

Reflexivity was embedded throughout data collection and analysis. The researcher kept brief reflexive notes after each interview, documenting initial impressions, emotional responses, and emerging ideas about how her dual practitioner–researcher role might be shaping interactions and interpretations. These notes, together with supervision discussions, were used to question overly positive readings of the data and to consider how participants’ ongoing relationships with the centre, and practitioners’ desire for a stronger evidence base for THR, might influence what was said. The researcher noted considerable variability in how forthcoming participants were. Some autistic participants provided minimal initial answers (for example, “yes,” “no,” “I don’t know”). Gentle probing sometimes yielded limited additional detail, and on reflection the researcher considered whether further elaboration of prompts could have helped. The researcher also reflected on recruitment challenges and on the decision to revise the minimum-session inclusion criterion from ten to three sessions. This informed a cautious approach to claims about typicality and long-term effects.

### 2.9. Method of Analysis

Data were analysed using reflexive thematic analysis (RTA; Braun & Clarke, 2019). This process enabled the researcher to engage deeply with the data and reflect on their interpretations. The analysis began with the researcher listening to the audio recordings while reading the corresponding transcripts to become immersed in the data and to begin noting initial analytic observations. Transcripts were then read multiple times and coded inductively using qualitative software, with codes capturing both semantic content (what participants said) and more latent meanings (how they positioned THR, themselves, and others).

Codes were iteratively reviewed, and those judged less relevant to the research questions were set aside. The remaining codes were then clustered into candidate themes and sub-themes, which were refined through repeated movement between coded data extracts, the full data set, and the developing analytic narrative. Throughout this process, the researcher maintained analytic memos and evolving theme maps to document key decisions, providing an audit trail of the analysis. Emerging themes were discussed in supervision to consider alternative readings and to ensure that they remained grounded in the data while acknowledging the inherently interpretive nature of RTA. Consistent with reflexive thematic analysis, inter-rater reliability was not sought; instead, rigour was pursued through depth of engagement, transparency of decision-making, and ongoing reflexive awareness of the researcher’s influence on the knowledge produced.

## 3. Results

The analysis generated five overarching themes, each with related sub-themes: (1) Facing the Puissance: barriers to accessing THR, (2) Pathways to Participation, (3) Embodied Engagement, (4) To Understand and To Be Understood, (5) Beyond the Arena—Impacts That Last.

### 3.1. Facing the Puissance: Barriers to Accessing THR

The term puissance refers to a high wall-jumping event in showjumping. Here, it is used metaphorically to capture the formidable, sometimes intimidating barriers that Autistic adults encounter when trying to access THR. Participants described a range of structural, systemic, and practical obstacles that made THR difficult to access or sustain. The metaphor of facing the puissance reflects how these barriers were experienced as high and sometimes daunting and challenging, but in some cases surmountable with the right support. Costs, funding, and finances were consistently described by both clients and practitioners as substantial barriers, alongside issues of physical and sensory accessibility and confusion about what THR actually involves. One participant emphasised this point directly:

“One of the barriers, like a really obvious barrier, is cost”.

(Participant 1, practitioner)

Much like the puissance, some barriers were challenging but ultimately surmountable, whereas other accessibility issues were viewed as more flexible, with solutions available to mitigate their effects. For example, Participant 2 (practitioner) described how busyness around the yard could be managed:

“If it is a bit busy, then the other staff tend to not like hang around the arena.”

Participants also shared some apprehension around starting THR “It’s quite scary. It’s a big thing to do” (Participant 8, client).

These accounts illustrate that while certain barriers are entrenched, others can be negotiated or adapted, reflecting the dynamic ways in which participants navigate participation. Within the overarching theme Facing the Puissance, such experiences informed the development of three sub-themes: Accessibility, Defining THR, and Finance and Funding. Together, these sub-themes highlight how practical, cognitive, and structural factors interact with participants’ engagement, shaping both the experience of THR and the extent to which clients can fully benefit from the service.

#### 3.1.1. Accessibility

The first sub-theme, Accessibility, explores participants’ experiences of Autistic adults navigating factors such as busy environments, physical abilities, and sensory needs. One consideration raised by practitioners was the physical needs of clients:

“Autistic adults that don’t have that same physical capability and would still benefit from it in terms of a mental health sense, but actually, we don’t have the set up for them to access it in a way we’d like, we can’t make them as safe”.

(Participant 1, practitioner)

Centres providing THR may not always have the appropriate facilities to meet these needs, creating a physical barrier, whether that’s safely assisting with mounting or simply accessing the yard area. Accessibility considerations also extended to sensory aspects of Autism. For example, when discussing typical riding school environments, Participant 2 (practitioner) shared what clients had previously told her:

“It’s too noisy, there’s too much going on, there’s shouting and you know, so that’s a big sensory overload.”

To address this, Participant 2 suggested offering quieter times, similar to “Autism-friendly hours” in supermarkets, as a way for riding schools to make THR more accessible for clients with heightened sensory needs.

The aforementioned examples illustrate how practical, environmental, and sensory factors impact on Autistic adults’ engagement with THR. Within the overarching theme Facing the Puissance, these findings show that navigating THR requires negotiating both physical and sensory challenges to fully benefit from the service. Taken together, these accounts show that access to THR is shaped not only by individual motivation but by the physical layout of centres, availability of appropriate equipment, and management of sensory demands. For Autistic adults in this study, quieter environments, flexible yard management, and attention to mobility needs were key to whether THR felt possible and safe.

#### 3.1.2. Defining Therapeutic Horse Riding

The second sub-theme, Defining THR, highlights how preconceptions, expectations, and lack of understanding of THR due to the difficulty in defining it, shaped early experiences for Autistic adults. Participant 1 (practitioner) reflected on how lack of definition leads to lack of clarity around what to expect:

“Because it’s really difficult to define. It’s quite niche…a lot of people confuse it with riding for physical health rather than mental health.”

Preconceptions often assumed THR would involve cantering, jumping, or controlling the horse, reflecting the broader equestrian culture.

“Preconception that it’s going to be about cantering around and jumping and making the horse do what’s expected…well, it is the culture in the equestrian world”.

(Participant 1, practitioner)

“when I had horse riding lessons before, like they would always tell me to be louder because the horse couldn’t hear me. So I was, I guess I was scared of that happening again. But then that made me quieter.”

(Participant 9, client)

Rigid thinking tendencies in Autistic adults could make adapting to THR challenging particularly for those who have previously ridden; however in Participant 1’s experience, once expectations were adjusted, participants reported rapid shifts in mindset, whilst this is considered a barrier, openness to change seems to be noticed by practitioners:

“Combined with the rigid thinking of autistic adults…once you can help them to do that, they’re very quickly then in a new mindset”.

(Participant 1, practitioner)

Participant 1 (practitioner), spoke about how some benefits especially around feeling, can be more challenging to measure and actually having a physical representation of progress, achievement and change may be beneficial to help clients to really recognise what has happened as a result of THR.

“We can talk about feeling more confident and feeling heard and feeling a sense of belonging, but that’s actually quite hard to measure and put your finger on.”

“Something like the rosettes…something you can hold that you’ve done and achieved.”

The suitability and responsiveness of horses were also central to defining THR and the ways in which it works, some conversation took place around the level of challenge that was relevant and acceptable for the horse to present to the client. Sourcing horses that fit this, proved harder for centres, but were perceived to have more benefit for the clients.

“Not any horse can be a therapeutic riding horse…horses that are willing to kind of attune to what the client’s needing in that session…probably has more benefit for the client.”

(Participant 1, practitioner)

Practitioners gave examples of ethical considerations for the horse and the adaptability of instructors, illustrating the intricate complexities revolving around the triad of practitioner—horse—client and ensuring all needs are safely and reasonably met.

“How can we genuinely help the horse to carry us and do that in a way that’s ethical and prioritises that relationship with them?”

(Participant 1, practitioner)

“there’s a lot of riding schools that offer therapeutic riding sessions. But I would question whether some of them are actually therapeutic riding sessions”.

(Participant 2, practitioner)

To provide some clarity to prospective clients about THR and their understanding of how the sessions may look, Participant 1 shared how she provides information on the website:

“We try and have it like a really solid description on the website of what it is and what people can expect.”

THR is often not clearly defined, or differs largely between centres and practitioners. Providing a clear explanation of what it is is not only beneficial to clients but also to practitioners and researchers who are exploring and delivering a cohesive version of THR. This subtheme illustrates how definitional ambiguity around THR can itself become a barrier to access. When Autistic adults and referrers confuse THR with sport riding or physical rehabilitation, expectations may not align with the more relational, emotionally focused work offered. Practitioners’ efforts to clarify what THR is, and is not, are therefore central to helping potential clients decide whether the service is right for them (Kerulo et al., 2020).

#### 3.1.3. Finance & Funding

The significant role of cost in shaping access to THR for Autistic adults was addressed by all participants, sharing both systemic challenges and varying individual experiences of affordability. Practitioners primarily focused on the cost of running and evidencing a niche service, whereas clients spoke about the affordability of session fees and how these constrained how often they could attend. Participant 1 (practitioner) emphasised:

“Cost is the biggest barrier to therapeutic riding…particularly in terms of winning funding because it doesn’t fit very neatly into therapy and counselling…and it also doesn’t fit into riding in terms of sport and physical activity, which is then a huge barrier to winning funding.”

Even when funding is secured, additional challenges arise.

“I just guess that a lot of that actually just relies on funding.”

(Participant 2, practitioner)

“Even when we do have funding, the reporting requirements are massive. So then we’re not likely to renew the funding, which means we’re going to have to put our costs back to the standard price.”

(Participant 1, practitioner)

Clients were acutely aware of financial limitations, particularly when costs impact frequency of attendance.

“I would do it every week if I could…But if there’s help out there to support, then probably you could get more because I’ll have it once any other week instead of once every month.”

(Participant 7, client)

Despite the challenges, some clients expressed that current pricing was manageable, relative to the benefits they experienced.

“I’m putting the bridle on. I’m learning lots of different things…So I think it’s a good price.”

(Participant 6, client)

Practitioners acknowledged that even small costs could disproportionately affect clients who are inherently disadvantaged:

“If people don’t have any spare money, £25 a month is between like a food shop for one person or not…there is definitely a huge amount of people who would love to come weekly but can’t afford it, so they come fortnightly or monthly…with any therapeutic intervention, the more consistent and the more regular …, the more effective it is, so it’s naturally less effective for them”.

(Participant 1, practitioner)

Systemic constraints were also reflected in the time and resources required to make THR measurable and tangible, such as producing rosettes for clients to recognise their achievements as previously mentioned in Defining THR.

“the amount of time, like unpaid time that goes into that then becomes another barrier…we don’t exactly have a lot of funding…very systemic issues, a vicious cycle”.

(Participant 1, practitioner)

Finance and funding are not simply individual barriers but are embedded within systemic and structural challenges faced by clients and providers alike. Within the overarching theme Facing the Puissance, these findings demonstrate how practical and financial realities shape access, engagement, and the effectiveness of THR, particularly for a client group that may be economically disadvantaged. While some clients find the current costs manageable, others are constrained in how frequently they can participate, which may limit the therapeutic impact. Overall, finances and funding operated as barriers at multiple levels. For clients, even relatively low fees could mean choosing between THR and essential expenses. For providers, limited and short-term funding streams, coupled with heavy reporting requirements, made it difficult to offer subsidised sessions consistently. These intertwined pressures meant that those who might benefit most from THR were often least able to attend regularly.

### 3.2. Pathways to Participation

#### 3.2.1. Opening the Gateway

Participants’ experiences of initial access to THR highlighted a number of factors that either facilitated or impeded engagement, including ease of booking, clear communication, predictable scheduling, and staff patience. Accessible systems and supportive practices helped clients to engage from the outset. For example, initial assessments that did not require riding but instead focused on explaining what to expect gave clients a chance to understand how future sessions might look. Although online booking is useful, flexibility in this process was also appreciated:

“Initial assessment where they don’t necessarily ride…have a chat about what to expect…that’s quite helpful as well.”

(Participant 1, practitioner)

“it’s easy to get in touch with us in whatever way feels safest.”

(Participant 4, practitioner)

“reminders and things are a massive help…also offers you the predictability…you know you’ve got your appointment.”

(Participant 5, client)

“I just find it better to book in person because then it’s done.”

(Participant 7, client)

Practitioners shared how THR was intentionally structured to support accessibility and inclusion noting the consideration of neurodivergent and mental health needs from the programme’s foundations:

“Very much geared for them from the start…we are going to be welcoming clients who maybe don’t see the world in the way we all do, but might have different needs.”

(Participant 1, practitioner)

Accessibility was further enhanced by practical considerations such as staff patience in delivering instructions and feedback. The supportive, inclusive environment provided by practitioners and the centre as a whole was also a defining feature of THR.

“I really…think it is a very accessible and inclusive place…being patient and allowing extra time…just that kind of kindness.”

(Participant 5, client)

“Guidance I’ve had has been really nice because as I say, I might forget. Okay. And it’s nice to just repeat it.”

(Participant 6, client)

Collectively, these accounts share how THR providers create an environment that actively opens the gateway for participation. By addressing practical, cognitive, and social needs, the programme facilitates engagement for Autistic adults, ensuring that initial barriers do not prevent them from experiencing the therapeutic benefits of riding. In combination, these practices “open the gateway” to THR by reducing uncertainty and practical effort at the point of first contact. For the Autistic adults in this study, straightforward booking routes, consistent reminders, and a visibly welcoming ethos were not incidental details but core elements of what made the service feel reachable and worth trying.

#### 3.2.2. Predictably Unpredictable

Participants shared experiences of consistency and unpredictability within THR, the careful balance between routine and variability that influences engagement and therapeutic benefit. Clients emphasised the importance of a predictable environment in supporting their participation and consistency of their attendance and how changes can be made easier by the predictability.

“It’s so easy to get overwhelmed…you end up not doing things or…having to let go of things. But I mean, how long have I been coming here? Over 18 months now. The fact that I’ve consistently still come here.”

(Participant 5, client)

“I was the only one to turn up because it was snowing, happily I’m here”.

(Participant 10, client)

“Even if the horses change, I will still be doing the same thing every time. So that really helps.”

(Participant 5, client)

Practitioners shared the careful management of predictability, noting that consistent routines, familiar horses, and a small, stable team all contributed to a safe and supportive environment.

“But there’s something about the consistency of that being true in terms of someone can come every time and having the same sort of experience…even if it’s more difficult some weeks, there’s always that environment that supports them to still achieve those things”.

(Participant 1, practitioner)

“I like to start each session by talking with the rider about how they’re feeling, how their week has gone, and how they’re feeling about the session ahead.”

(Participant 3, practitioner)

“Horses in therapeutic riding in general, you can sort of manage an unpredictable horse because the focus is on the communication and relationship with the horse and that’s OK. But in my experience, autistic adults have found that a little bit harder to deal with just because if you don’t quite know what you’re coming to…even if you’re riding the same horse, you don’t know how that horse is going to feel”.

(Participant 1, practitioner)

Too much unpredictability could also shift the focus away from the client, reducing the therapeutic benefit:

“When the instructors just have to manage the horse because the horse has become unpredictable or difficult, that actually then the focus is taken away from the client. Clients can deal with that to an extent, but at some point, it becomes quite damaging”.

(Participant 1, practitioner)

For Autistic adults, predictable routines and familiar environmental cues provide a sense of safety, while unpredictable elements, particularly relating to horse behaviour, require careful management by practitioners. Within the overarching theme “Pathways to Participation”, “Predictably Unpredictable” highlights the nuanced interplay between stability and variability, illustrating how delivering THR in a way that provides a level of predictability for the client allows them to safely engage with unpredictability which is something that Autistic adults may usually be averse to. This subtheme highlights the delicate balance between routine and variability in THR. Predictable structures, familiar horses, and consistent staff made it possible for Autistic adults to keep attending, while carefully managed, low-level unpredictability offered opportunities to practise coping with change. When unpredictability became too great, however, the focus shifted away from the client and therapeutic benefits were weakened.

#### 3.2.3. Tailored and Personalised

The importance of instructors’ ability to adapt THR to meet the individual needs of clients is described by participants sharing how personalised approaches, both in communication and environmental structuring, made participation more accessible and meaningful. Processes such as meeting people at the gate and having clear guidance contributed to predictability and reduced anxiety:

“spent some time on the website and read that…and it gave a very warm and friendly feel to the place, which is always important to me…Just the way something’s written and the vibe it kind of gives matters. So it felt very friendly.”

(Participant 5, client)

“I just came down here and it’s also good that you meet people at the gate…all of that worked very well for me, in terms of…finding it and accessing it on the first appointment.”

(Participant 5, client)

Practitioners described being able to actively adapt sessions to clients’ communication preferences, sensory needs, and learning styles; clients also reiterated practitioners’ ability to do this.

“things still need to be repeated a bit more?…That repetition was actually very helpful…It helps me…remember what I’m doing.”

(Participant 6, client)

“you’re inclusive of people with different communication styles, and I think that’s the most important thing”.

(Participant 9, client)

“Guess the way I structured my sessions would be I asked the client what they need. I asked the client how and where they prefer to be, how they prefer to communicate, what sensory issues they may have… I will always try to adapt as best as I can.”

(Participant 2, practitioner)

“Actually when when they’re in the riding sessions and we’re able to adapt the sessions and facilitate those choices … those additional needs are met.”

(Participant 4, practitioner)

“We adapt to clients that need more black and white language and more clear-cut instructions…but very rarely has the client to teach us anything in terms of what they need.”

(Participant 1, practitioner)

Tailoring both the physical and procedural elements of THR is hugely impactful for supporting engagement among Autistic adults. By prioritising individual needs, THR providers create an environment in which clients can confidently participate, learn, and experience the therapeutic benefits of the sessions. Structured flexibility allows clients to feel understood and supported, enhancing the accessibility and effectiveness of THR. These accounts emphasise that tailoring is not an “add-on” but central to making THR usable for Autistic adults. Adjustments in language, pacing, meeting arrangements, and sensory input allowed clients to feel understood and in control. For participants here, feeling that the service would flex around their needs was a precondition for engaging with the more challenging aspects of the work.

### 3.3. Embodied Engagement

Embodied Engagement explores how THR enables Autistic adults to experience their bodies as sites of awareness, tuning in to physical sensations and movement. Through multisensory processes and the unique movement of the horse, participants described reconnecting with bodily sensations and finding space for emotional release, alongside moments of enjoyment and belonging.

#### 3.3.1. Reconnect & Release

Participants repeatedly emphasised the multisensory nature of THR, and its capacity to draw attention to the body. Participants described how grooming and riding provided a rich, positive sensory environment, creating a present-moment awareness, which was an important step to becoming ready to ride.

“That responsibility to be present in the moment to ride gives like a purpose to that calming.”

(Participant 4, practitioner)

“On a sensory level…the smell of the horses, the different textures of their coats…some of them are softer than others, that lovely warm snuggly bit underneath their mane.”

(Participant 5, client)

“tune into body, tune into the horse’s body, tune into how the two are interacting,”

(Participant 5, client)

Participant 7 described how mounted mindfulness practices fostered physical release:

“Walking around and then at the end we’d stop…deep breath, heavy breath…mind emptying…and you can feel it falling out and coming out…It’s like you just pull the plug out of the bath…all the heaviness has just dropped out of you…you’re ready for the next battle.”

“I think it, it gives me like a purpose to sort of, leave the house and go somewhere where there’s other people…”.

(Participant 8, client)

Practitioners intentionally created the conditions for this somatic reconnection, ensuring the space held was safe for the client to do so.

“We brush the horse, we chat about how we’re feeling, how the horse may be feeling…then we get on when the client feels they’re happy…it feels very natural and relaxed.”

(Participant 2, practitioner)

“Many of these adults have learned to disconnect from the body because it feels overwhelmed all the time. This gives them a space where they can come back to connecting with their body in a really safe way…huge outpouring of emotion…in a really positive way, kind of released a lot of that.”

(Participant 1, practitioner)

Here, the horse’s rhythmic movement and the supportive environment together create a safe and contained space in which Autistic adults can re-inhabit their bodies and release accumulated tension, a process that may be difficult to access in more conventional therapeutic settings. Overall, “Reconnect & Release” captures how THR provided a structured opportunity to tune into bodily sensations in a way that felt safe rather than overwhelming. Through grooming, riding, and guided reflection, Autistic adults described becoming more aware of their own bodies and emotions and experiencing moments of relief from accumulated tension that could be hard to access in more traditional, verbal therapies.

#### 3.3.2. Unbridled Joy

Alongside quiet release, participants also described moments of spontaneous pleasure and playful freedom. Participant 5 captured the “instant sense of happiness” she felt on arriving and “watching the horses and seeing them and catching up on what they’ve been up to.”, for some, this instant impact could be seen once on the horse.

“feel quite calm when I’m on horses,”

(Participant 6, client)

“quite a few clients, once they get on the horse, or even once they’re just with the horse, appear to relax a bit more…they appear to seem happier within themselves around the horses.”

(Participant 2, practitioner)

Participant 1 highlighted that within this atmosphere of acceptance, clients “can thrive, they enjoy it…they can unmask and just relax into that relationship”.

Participants suggest that THR offers more than therapeutic release: it provides opportunities for unguarded and unmasked enjoyment, for reconnecting with a child-like sense of play, and for experiencing belonging and self-expression without social demands.

“it kind of is healing in that way if that’s not too cheesy to say, your younger self just getting back in touch with that and doing silly things and taking all the adult stresses off”.

(Participant 1, practitioner)

The theme Embodied Engagement shows how THR supports Autistic adults to engage with their bodies in ways that are at once both restorative and liberating. By combining mindful, sensory attuning with the simple pleasure of being with horses, THR creates an embodied therapeutic experience where participants can both let go of tension and rediscover the joy of inhabiting their own bodies. Alongside calmer states, participants described THR as a source of uncomplicated pleasure and playfulness. These moments of “unbridled joy” (e.g., arriving at the yard, spending time with horses, and temporarily setting aside everyday pressures) contributed to well-being in their own right and sat alongside more explicitly therapeutic processes such as emotional release or insight.

### 3.4. To Understand and to Be Understood

Participants discussed the importance of authentic relationships and relational reciprocity within therapeutic horse riding (THR), “having time to experience the relationship either with the horse or with myself or both.” (Participant 4, practitioner). Central to this theme was the experience of both understanding and being understood by the horse, the instructor, and oneself. Participant 8 (client) noticed this was possible with guidance despite previously having negative experiences, “I’ve always been told that I’m not very good at reading body language, but actually I can read their body language when I know what I’m looking for I can”.

#### 3.4.1. Authenticity in Relationships

Participants highlighted how authentic, reciprocal connections with both horses and practitioners were important to their therapeutic experience. Clients described the process of cultivating these bonds as one that required patience and gentle persistence; initial anxieties about whether a horse would “like” them often gave way to recognition of the horse’s individuality and communication style. As they learnt to read cues and respond accordingly, they began to experience the relationship as mutual “they know me and I know them”(Participant 5, client) rather than one-sided. One client described her evolving relationship with “Holly” (a mare), illustrating how patience and gentle persistence helped transform initial anxiety into mutual trust.

“Oh no, she doesn’t like me, you know?’…patience had to come into…that kind of cultivating that relationship with Holly (mare) and then a lot of gentleness.”

(Participant 5, client)

“You know, trying to learn her way of communicating so that that could help our relationship, connection.”

(Participant 5, client)

“I’m able to understand horses a little bit better. I’m able to understand their behaviour. Yeah. Because no horse is the same”.

(Participant 10, client)

Clients were often able to recognise the two-way process between the horses’ experiences and their own, facilitated by practitioners creating opportunities for reflection, empathy and connection. Helping clients both understand and feel understood by the horse was described as a deliberate aim of the sessions.

“that relationship is really nice.”

(Participant 6, client)

“It can be quite a nice two-way process for the client and the horse to understand each other a bit better”.

(Participant 2, practitioner)

“But when you get on there…you’re concentrating on what you’re doing…It’s just you two, you and the horse.”

(Participant 7, client)

These developing relationships brought an emergent self-awareness and agency that extended beyond the riding arena. Working in real time with a responsive animal requires clarity and presence, requiring them to “find a way” rather than rely solely on verbal communication. For some, this fostered confidence and even a sense of discovering a “new me,” suggesting an evolving self-concept through the embodied experience.

“You can talk about it endlessly in therapy and actually therapeutic riding makes…it happening in the here and now, and you’re going to have to deal with it and find a way.”

(Participant 1, practitioner)

Learning to communicate effectively with the horses was experienced as both experiential and embodied. Clients described a growing competence in giving clear instructions and recognising the horse’s feedback, which practitioners interpreted as evidence of developing leadership and relational attunement. This physical, here-and-now engagement allowed therapeutic insights to be enacted rather than merely discussed, reinforcing a felt sense of efficacy and mutual trust.

Clients described the significance of building genuine connections with the horses and practitioners, emphasising patience, gentleness, recognition and learning about the horses as catalysts to creating meaningful relationships.

Clients also valued the experience of understanding the horse’s behaviour and adapting their communication accordingly, which was central to feeling effective and competent.

“So it was understanding that…the instructions that I was giving needed to be quite clear. So it understood me and what I wanted to do with it.”

(Participant 6, client)

“What do they want to do? What do they not want to do? Are they tired? Are they in pain?”

(Participant 10, client)

“Found that kind of sense of leadership in herself…Holly (mare) did listen, and she was effective. And then she was able to work through it.”

(Participant 1, practitioner)

In sum, this subtheme illustrates how authentic, reciprocal relationships with horses and practitioners enabled Autistic adults to experiment with new ways of communicating, leading, and being with others. Clients’ growing sense that they were both understanding and understood by the horse seemed to support emerging feelings of competence, agency, and “a new me” beyond the arena.

#### 3.4.2. Herd, Held and Supported

The sense of being heard, held, supported, and safe within the THR environment was created within the context of the relationships, as well as the physical space—which some described as a “bubble”, offering a pressure-free and anxiety-reducing space.

“Even as soon as you pull into the car park, it’s like this bubble.”

(Participant 5, client)

“When I come here…all the stress…you get in the driveway, it’s like…the whole place is like…a bubble.”

(Participant 7, client)

This slow, gentle, and predictable space helped clients feel safe and supported, enabling engagement at their own pace.

“there’s no hurry to this session, the whole session is very slowly done…very much at that person’s pace at the horse’s pace as well.”

(Participant 2, practitioner)

“If you can just sit there and be at one with the horse and understand the horse, you can get through the day.”

(Participant 7, client)

Together, these accounts suggest that THR offered more than a series of sessions. It provided a containing “bubble” in which clients felt held by the environment, the herd of horses, and the human staff. The combination of predictable pacing, attuned relationships, and a low-pressure atmosphere allowed Autistic adults to drop their guard, contributing to a felt sense of safety that underpinned other therapeutic gains.

### 3.5. Beyond the Arena: Impacts That Last

Participants described how THR produced effects that extended beyond the immediate session, highlighting the enduring influence of the relational, emotional, and practical experiences they had with horses and practitioners. These impacts were felt in clients’ daily lives, contributing to confidence, well-being, and a sense of competence, demonstrating that the benefits of THR are not confined to the arena.

#### 3.5.1. Carried by Connection

A central aspect of lasting impacts was the connections formed with both horses and instructors. Participants described these relationships as reciprocal and emotionally sustaining, providing a sense of support that could be carried into everyday life, reinforcing that clients have the autonomy to communicate their needs, and ask for them to be met.

“The joy as well of connecting with them…reading their placement of their body, how they communicate to you.”

(Participant 5, client)

“Communication skills improve too, both verbal and non-verbal.”

(Participant 3, practitioner)

“What the horse might be communicating to them…helping them to also communicate their needs better.”

(Participant 2, practitioner)

These relationships also fostered a sense of belonging and continuity, built around the consistent and containing experiences provided by the centre environment, horses and practitioners.

“They’ve become part of the centre…, it’s like that real sense of belonging…to be able to come in, know all the horses and find out how they’ve been doing…and have that on a long-term basis.”

(Participant 1, practitioner)

“I like the therapeutic side because I get to groom them, I get to take them out…they really know me now and they know me as a rider.”

(Participant 6, client)

This subtheme shows how relationships developed in THR were experienced as emotionally sustaining and portable. Clients and practitioners alike described how learning to recognise and express needs in the arena, and having those needs responded to, supported clients to communicate more effectively and to seek support in other areas of life.

#### 3.5.2. Every Single Time

Clients reported that the positive effects of THR were consistent and cumulative, with each session building on previous gains and contributing to ongoing feelings of confidence and well-being

“I feel the benefits every single time I come…it drips into my week and that’s important.”

(Participant 5, client)

“I can volunteer…that’s given me more confidence in myself and the group sessions…I’m finding the new me.”

(Participant 7, client)

“You can see they feel and see their confidence grow while they’re around the horses…the enormous amount of well-being I see within the clients.”

(Participant 2, practitioner)

The repetition and continuity of sessions reinforced learning and self-assurance, demonstrating that THR provides sustained rather than transient benefits, along with transferable skills that can be felt within daily living contexts.

“A little bit like happier as they leave. Maybe a little bit more relaxed or even kind of proud of themselves for giving them that space for a bit of self-care and time for themselves as well.”

(Participant 4, practitioner)

“Every Single Time” captures participants’ sense that the benefits of THR were reliable and cumulative. Rather than isolated “good sessions”, clients described a steady accrual of confidence, self-care, and willingness to try new roles (such as volunteering), suggesting that, for this group, repeated participation produced changes that extended into daily routines and self-concept.

#### 3.5.3. Skills in Practice

THR facilitated practical skill development, from riding techniques to horse care, which translated into real-life competence and personal growth.

“You’re responsible for this horse at that moment…but I never felt unsafe.”

(Participant 5, client)

“grooming, checking the horse, picking their feet out…they’re learning all about horse care as well.”

(Participant 2, practitioner)

“I’ve been able to apply that to different things…take what I’ve learned and use it in other places in my life.”

(Participant 6, client)

THR appears to function as a self-efficacy laboratory in Bandura’s (1977) terms. Through developing skills of riding and horse-care tasks, clients met challenges, from picking out a horse’s feet to guiding the horse safely, within a scaffolded context of safety, support and acknowledgements of those achievements.

“At the end, we always talk about what went well and I make sure to praise any achievements, big or small.”

(Participant 3, practitioner)

“There’s usually like multiple, like small achievements throughout the sessions as well, which seems to kind of help people feel a little bit better. They’ve achieved something.”

(Participant 4, practitioner)

These experiences foster a belief in personal competence that participants report transferring to other life domains, for example asking things of others, which previously may have felt uncomfortable.

“recently they have spoken about how they’ve been able to go out and ask for someone to maybe do something for them or express their needs a bit more confidently.”

(Participant 4, practitioner)

In turn, the act of caring for the horse provides a vicarious model of care and responsibility, reinforcing an emerging narrative of themselves as capable and self-caring. Thus, the THR setting cultivates not only practical skill but the psychological mechanism of self-efficacy that underpins adaptive functioning and personal growth.

“So I think from being more assertive, I think it’s helped with my confidence…I do have the confidence to be able to see lots of different things.”

(Participant 6, client)

“By caring for the animals, they’re also learning to care for themselves better…taking care of themselves is also very important.”

(Participant 2, practitioner)

Overall, this subtheme illustrates how THR functioned as a kind of self-efficacy laboratory. Within a scaffolded environment, Autistic adults practised concrete skills, experienced success, and received explicit recognition for their achievements. Participants and practitioners linked these experiences to increased assertiveness and self-care in other domains, although we remain cautious about generalising beyond this self-selecting, verbally fluent group.

## 4. Discussion

This study explored how Autistic adults and THR practitioners understand the benefits, barriers, and broader life impacts of THR delivered as a mental-health-oriented equine-assisted service (EAS) at a single UK centre. Using semi-structured interviews and reflexive thematic analysis (RTA), we constructed five themes: *Facing the Puissance: barriers to accessing THR, Pathways to Participation, Embodied Engagement, To Understand and To Be Understood, and Beyond the Arena—Impacts That Last* (see Table 1). In line with the aims of qualitative inquiry, the goal was to offer an in-depth, contextualised understanding of how THR is experienced by a specific group of Autistic adults and practitioners, and explore the benefits, barriers and broader impacts of participation in THR for autistic adults. By foregrounding the perspectives of both clients and practitioners, this research contributes to a limited evidence base that has historically prioritised the views of carers or focused on children and adolescents (McDaniel Peters & Wood, 2017; Warner et al., 2022). The findings highlight the therapeutic potential of THR as an embodied, relational, and accessible intervention while also revealing significant physical and systemic barriers that limit its potential reach.

### 4.1. Summary of Main Findings

Autistic adults in this study described THR as a valued source of enjoyment, calm, and confidence, with perceived benefits that accumulated over time and, for some, extended into everyday life (e.g., increased willingness to leave the house, volunteering, and asking others for support). These accounts are consistent with research showing that equine-assisted programmes can enhance self-esteem, self-efficacy, and psychological well-being in other populations, including young people experiencing social and emotional difficulties and adults with psychiatric diagnoses (Bizub et al., 2003; Burgon, 2011; Cantin & Marshall-Lucette, 2011; Karol, 2007; Hatcher et al., 2019). Participants highlighted three interlinked features as central to THR’s perceived value: (a) an explicitly structured, predictable environment that accommodated sensory and communication needs; (b) authentic, reciprocal relationships with horses and practitioners; and (c) opportunities to practise leadership, communication, and self-advocacy in a scaffolded, embodied way. These findings complement and extend the largely child-focused equine-assisted autism literature, which has identified improvements in social communication, adaptive behaviour, and emotional regulation (Gabriels et al., 2015; Cleary et al., 2024; Xiao et al., 2023; Browne et al., 2025), by foregrounding Autistic adults’ first-person perspectives on similar processes in adulthood.

At the same time, both clients and practitioners emphasised substantial barriers to accessing and sustaining THR, particularly costs, definitional ambiguity, and limited facilities and staffing. These barriers mirror broader structural inequities experienced by Autistic adults in healthcare and employment (Howlin & Magiati, 2017; UK Parliament, 2023; Harmuth et al., 2018), and sit alongside longstanding concerns about the cost, availability, and standardisation of equine-assisted services (Bachi, 2012; Kendall et al., 2014; Lentini & Knox, 2009; Seery & Wells, 2024).

### 4.2. Relational Dynamics and Embodied Engagement as Therapeutic Mechanisms

Across themes, participants located much of THR’s perceived therapeutic value in relational dynamics among horse, client, and practitioner. Autistic adults described feeling “heard,” “held,” and “understood” within the THR environment, and emphasised the importance of trust and reciprocity in their relationships with horses and instructors. These relational dynamics were perceived as contributing to emotional regulation, enhanced self-awareness, and increased confidence. These findings align with previous research emphasising the relational basis of equine-assisted interventions, where non-verbal communication, mutual responsiveness, and attunement form the foundation of therapeutic outcomes, which support emotional regulation and a sense of being “met” by another (Bizub et al., 2003; Burgon, 2011; Gabriels et al., 2015; Karol, 2007; Ward et al., 2022).

The human–horse relationship appears to offer a unique context for self-exploration and growth as well as for practising leadership, boundary-setting, and clear communication. Horses’ sensitivity to emotional states provides immediate feedback, fostering self-regulation and social-emotional learning (McCrea et al., 2025). Autistic adults described learning to give unambiguous cues, interpret horses’ responses, and adjust their own behaviour accordingly, often with practitioners explicitly framing this as leadership or collaborative problem-solving. This echoes studies highlighting horses’ sensitivity to human affect and behaviour and their role as responsive partners in therapeutic work (Hatcher et al., 2019; Anastasya et al., 2024; Ward et al., 2022), as well as theoretical accounts that emphasise co-regulation and embodiment as mechanisms of change in equine-facilitated psychotherapy/learning (Karol, 2007; Garcia, 2013). In the current study, these dynamics extended beyond the horse-client relationship to include the practitioner, findings which were also seen within Browne et al.’s (2025) study where children expressed the kindness and safety felt when with their practitioner.

Participants also emphasised embodied engagement as central to their experience. Grooming, riding, and simply being in close proximity to horses created a multisensory environment in which Autistic adults reported “tuning into” their bodies, noticing tension and release, and experiencing both calming and “unbridled” enjoyment. These narratives resonate with broader evidence that equine-assisted activities can foster bodily awareness, self-confidence, and empowerment (Bizub et al., 2003; Burgon, 2011; Cantin & Marshall-Lucette, 2011), and with literature on embodied therapies that conceptualise bodily experience as central to emotional processing and trauma integration (Fuchs & Koch, 2014). For some clients, THR functioned as a “self-efficacy laboratory”: within a safe, scaffolded context, they encountered concrete challenges (e.g., mounting, riding independently, caring for the horse), succeeded, and had those achievements explicitly recognised. This maps closely onto Bandura’s (1977) account of self-efficacy, whereby mastery experiences and positive feedback strengthen beliefs in one’s own capabilities, and is consistent with reports that equine-assisted interventions can enhance confidence and agency in both Autistic and non-Autistic populations (Hatcher et al., 2019; Ward et al., 2022; Browne et al., 2025).

### 4.3. Environmental Structure, Predictability, and Engagement

The themes *Pathways to Participation* and *Embodied Engagement* highlight the centrality of environmental structure and predictability in enabling Autistic adults to access and sustain THR. Participants valued clear information about what sessions would involve, straightforward booking routes, reminder systems, and initial assessments that did not require immediate riding. Within sessions, consistent routines, familiar staff and horses, and clearly defined expectations created a safe and contained therapeutic space, reducing anxiety and supporting sustained participation. These results are consistent with literature for autistic children accessing THR which emphasised the need for adaptable, sensory-aware environments and flexible approaches to communication (Browne et al., 2025; Cleary et al., 2024). The findings are also consistent with broader literature on autism and service accessibility, which emphasises the value of predictability and routine in reducing cognitive load and sensory overwhelm (Howlin & Magiati, 2017; Lord et al., 2018; Pellicano et al., 2022).

Interestingly, a degree of unpredictability, such as minor behavioural variations in horses, was also described by the practitioners as therapeutically beneficial when managed carefully. This aligns with the concept of “graded exposure” within autism support, where safe encounters with variability can promote resilience and flexibility (Langdon et al., 2024. However, when unpredictability increased beyond a certain threshold—such as in busy yard environments or when horses became highly reactive—participants felt that the focus shifted away from the client and therapeutic benefits diminished, underscoring the need for tight environmental and ethical management in THR settings (Bachi, 2012; Ward et al., 2022). Practitioners must therefore strike a careful balance, creating environments that are structured enough to support participation but flexible enough to allow for growth.

### 4.4. Accessibility, Equity and the Challenge of Systemic Barriers

The theme *Facing the Puissance* foregrounds systemic, structural, and practical barriers that constrain Autistic adults’ access to THR. The puissance metaphor captures how these barriers were experienced as tall, sometimes intimidating obstacles that had to be negotiated before any potential benefits could be realised. Financial barriers were salient at both client and provider levels. Clients described weighing THR fees against essential expenses and, in some cases, reducing the frequency of sessions, with potential implications for the continuity and depth of therapeutic work. Practitioners highlighted the difficulty of sustaining a resource-intensive, labour-heavy service for a socio-economically disadvantaged client group in the context of short-term funding cycles and substantial reporting requirements. These accounts are consistent with evidence that Autistic adults face disproportionate unemployment and economic hardship (Buescher et al., 2014; Harmuth et al., 2018; Farkas et al., 2021) and with findings that equine-assisted interventions are often expensive to deliver and poorly integrated into mainstream funding systems (Burgon, 2011; Kendall et al., 2014; Bachi, 2012). Specifically, due to its hybrid nature as both a therapeutic intervention and a recreational activity, THR is frequently excluded from mainstream funding streams. This lack of institutional recognition not only limits affordability but also contributes to inconsistent provision and variable quality across centres (Bachi, 2012; Kendall et al., 2014).

Participants also pointed to definitional ambiguity as a barrier. Potential clients and referrers frequently conflated THR with sport riding or physical rehabilitation, complicating decisions about suitability and funding. This mirrors long-standing concerns that inconsistent terminology and heterogeneous practice models within equine-assisted interventions impede both research and commissioning (Kerulo et al., 2020; Lentini & Knox, 2009; McDaniel Peters & Wood, 2017; Ward et al., 2022). Recent consensus work in the United States has recommended adopting “equine-assisted services” (EAS) as an umbrella term, with specific labels (e.g., THR, equine-assisted psychotherapy) reserved for clearly defined practice models (Wood et al., 2021). Situating the present THR programme within this EAS framework may aid comparison with other studies and support clearer communication with commissioners and clients about what is being offered and evaluated.

These intertwined economic and conceptual challenges suggest that, without structural changes in funding and regulation, THR is likely to remain accessible only to a small subset of Autistic adults with sufficient financial and logistical resources, despite potential benefits. This sits uneasily with policy concerns about inequities in access to healthcare and support for Autistic people and those with learning disabilities (NICE, 2025 UK Parliament, 2023; Jones et al., 2025).

### 4.5. Methodological Strengths and Limitations

Several strengths of the study enhance its contribution. First, it focuses explicitly on Autistic adults, a group underrepresented in both autism and EAS research, where children and adolescents often viewed through caregiver or practitioner lenses predominate (McDaniel Peters & Wood, 2017; Warner et al., 2022; Botha & Cage, 2022). Second, the interview guide was co-developed with Autistic university students from the Progressive Autism Group, colleagues at the Autism Research Innovation Centre, and experienced THR practitioners, supporting content validity, alignment with Autistic priorities, and sensitivity to clinical realities (Kallio et al., 2016; Holt et al., 2022; Moore et al., 2024). Third, the guide was shared in advance and is provided in full as Supplementary Materials, reflecting best practice for accessible research with Autistic people (Botha & Cage, 2022; Jones et al., 2025). Finally, the use of RTA allowed for a rich, interpretive engagement with the data and explicit attention to the researcher’s dual practitioner–researcher role (Braun & Clarke, 2013, 2019). However, several limitations must be acknowledged. The sample may reflect a participant group with relatively positive experiences, as all Autistic participants had continued to engage with THR. Furthermore, all participants were recruited from a single THR centre, sharing a specific organisational culture and way of conceptualising THR. This single-site design enhances contextual specificity but limits transferability to other settings or practice models (Bachi, 2012; Ward et al., 2022). Recruitment challenges also highlight potential selection bias, as individuals with negative or neutral experiences may have been less inclined to participate. Also, all practitioners were invested in the programme and expressed a desire for THR to become more evidence-based, which may have encouraged them to emphasise positive aspects and under-report challenges, a well-recognised issue in practitioner-led evaluations (Bachi, 2012; Seery & Wells, 2024). Additionally, the dual role of the researcher as both practitioner and interviewer introduces the possibility of bias, although reflexive practices were employed to mitigate this, and a relationship that sought to foster open conversation was actively maintained.

The characteristics of the Autistic sample further constrain transferability. All Autistic participants were able to give informed consent and engage in semi-structured interviews, suggesting average or above-average intellectual and verbal functioning, but no formal diagnostic or psychometric data were collected to specify support needs or position participants within conventional severity levels. Experiences of Autistic people with higher support needs, co-occurring learning disabilities, or significant communication challenges may differ substantially, both in relation to THR and to service accessibility more broadly (Moore et al., 2024). The cross-sectional design also limits inferences about durability and causality. Participants were asked about short- and longer-term effects, but we did not track change over time or compare THR with alternative interventions. As in other qualitative work, themes represent the researcher’s interpretive reading of the data rather than objective categories, and different analytic lenses could have generated different emphases (Braun & Clarke, 2013, 2019). Taken together, these limitations mean that the findings should be treated as offering in-depth, situational insight, rather than generalisable evidence of THR’s efficacy across the Autistic population.

### 4.6. Implications for Practice, Policy & Future Research

The findings have several important implications for practice. First, they highlight the need for THR providers to adopt neurodiversity-informed approaches that prioritise: (a) predictable, clearly communicated structures for referral, booking, and attendance; (b) sensory-aware environments, including quieter yard times and minimisation of competing demands; and (c) proactive tailoring of sessions to individual communication preferences, sensory profiles, and goals. These suggestions are consistent with neurodiversity-affirming approaches to mental health care (Botha & Cage, 2022; Jones et al., 2025; Moore et al., 2024) and with emerging guidelines for autism-informed adaptations of therapeutic interventions more broadly (Moore et al., 2024). Additionally, practitioners should be mindful of the therapeutic balance between structure and variability, ensuring that sessions are both predictable and appropriately challenging.

In policy and commissioning terms, these qualitative findings alone are not sufficient to justify widespread integration of THR into UK healthcare and social care frameworks. Commissioning decisions must consider not only perceived efficacy but also cost-effectiveness, scalability, and comparative advantage over other interventions (Buescher et al., 2014; Rogge & Janssen, 2019; Zhao et al., 2023). THR is resource-intensive, dependent on specialist facilities, horse welfare, and highly trained staff, and is currently unevenly distributed (Hatcher et al., 2019; Ward et al., 2022; Seery & Wells, 2024). The present study suggests that, for a subgroup of verbally fluent Autistic adults accessing a particular kind of THR service, participation can be experienced as meaningful and helpful. However, robust evaluations, including economic analyses and comparative trials, are needed before THR can be responsibly positioned alongside, or within, mainstream services (Xiao et al., 2023; Cleary et al., 2024; Ward et al., 2022).

Future research can extend this work in several directions. Qualitative studies with more diverse Autistic samples, including people with higher support needs, alternative communication modes, and those who declined or discontinued THR, would help to clarify for whom THR is acceptable or beneficial (Moore et al., 2024; Warner et al., 2022). Mixed-methods and quantitative studies could explore specific outcomes such as anxiety, self-efficacy, and social participation over time, and compare THR with other interventions or activities (Gabriels et al., 2015; Xiao et al., 2023; Ward et al., 2022). Mechanism-focused research informed by theories of sensory processing, embodied cognition, and attachment or co-regulation could deepen understanding of how equine-assisted services exert their effects (Fuchs & Koch, 2014; Karol, 2007). Finally, studies of cost-effectiveness and sustainable service models would be particularly valuable in informing decisions about whether, and how, THR might be integrated into mental health provision in an equitable way (Buescher et al., 2014; Rogge & Janssen, 2019; UK Parliament, 2023).

## 5. Conclusions

This study contributes valuable new knowledge to an emerging area of therapeutic practice, shedding light on how Autistic adults experience and benefit from therapeutic horse riding within a mental-health-oriented programme. Autistic clients and practitioners described THR as a uniquely embodied, relational, and accessible approach to supporting mental well-being, one that can foster confidence, emotional regulation, and a sense of belonging that extends beyond the arena into everyday life. At the same time, participants highlighted substantial financial, logistical, and definitional barriers that restricted who could access and continue THR, raising questions about equity and sustainability in the current landscape of provision (Burgon, 2011; Kendall et al., 2014; Bachi, 2012). Within the constraints of a small, single-centre sample of verbally fluent Autistic adults, the findings suggest that THR can align with neurodiversity-affirming principles when intentionally designed with Autistic needs in mind, offering predictable, sensory-aware environments; privileging client choice and agency; and valuing diverse communication styles (Botha & Cage, 2022; Jones et al., 2025; Moore et al., 2024). Rather than presenting THR as a universal solution, we propose that it should be understood as a potentially valuable option for some Autistic adults in particular contexts. Realising its potential will require not only further research and close attention to horse welfare and practitioner training, but also critical engagement with issues of access, cost, and standardisation across the broader field of equine-assisted services (Kerulo et al., 2020; McDaniel Peters & Wood, 2017; Ward et al., 2022; Seery & Wells, 2024).

## Figures and Tables

**Table 1:** Themes and sub-themes produced from a reflexive thematic analysis of the data.

Main Themes	Sub-Themes
Facing the Puissance	Accessibility
barriers to accessing THR	Defining Therapeutic Riding
	Finances and Funding
Pathways to Participation	Opening the Gateway
	Predictably Unpredictable
	Tailoring and Personalisation
Embodied Engagement	Reconnect and Release
	Unbridled Joy
To Understand, and To Be Understood	Authenticity in Relationships
	Herd [SIC], Held, Supported
Beyond the Arena—Impacts That Last	Carried by Connection
	Every Single Time
	Skills in Practice

## Data Availability

The data presented in this study are available on request from the corresponding author due to privacy of the participants who continue to attend sessions.

## Supplementary Materials

The following supporting information can be downloaded at: https://www.mdpi.com/article/10.3390/bs16010084/s1, Interview Schedule.

## Abbreviations

The following abbreviations are used in this manuscript:

EAS	Equine Assisted Services
THR	Therapeutic Horse Riding
