# Supplementary material for: Perceived Benefits and Barriers for Autistic Adults Accessing Therapeutic Horse Riding for Mental Health
Source: Behav Sci (Basel). 2026 Jan 7;16(1):84. doi: 10.3390/bs16010084 (PMC12837896; doi:10.3390/bs16010084)
Supplement: Supplementary file 1 [file behavsci-16-00084-s001.zip › behavsci-3989751-supplementary.pdf]

<u>Interview Schedule</u>

<u>Autistic Adult</u>

Can you start by telling me how you first learnt about and accessed therapeutic riding for mental health?	Aims to understand how it was accessed e.g. social media, referral,	
How was your experience of finding and beginning sessions?	Novel question – how was the experience – how does this relate to the benefits if it was difficult to access, was it worth it?	
Please outline any barriers faced	Understand barriers faced, do these align with barriers to providing THR such as cost? Are they common barriers for adults with autism accessing any therapeutic environment or are these specific to THR	Kendall et al., 2013 – expensive to run… is it expensive to access?
How could these barriers be improved or removed	Open ended and opinion based – think about how these can be applied to services in the future	
Are there any short term impacts either positive or negative from attending THR	Immediate benefits between start and end of the session – encourage a narrative answer such as examples within sessions	Boyd & le Roux (2017) – continuing happiness once on the horse & focus
Are there any long term impacts either positive or negative from attending THR	Effects noticed outside of sessions and throughout sessions for example impacts of social experiences or impact on confidence across sessions	Boyd & le Roux (2017) - The bond between the horses and the children was perceived as aiding the children in obtaining more benefit out of the activity; development of social and behavioural skills
Does THR affect the way you deal with other challenges you face?	Open ended and non directive? Prev lit suggests new skills applicable to life, mostly focused on social skills, what else is there?	Boyd & le Roux (2017) development of social and behavioural skills
Wider impacts	Unsure if this is relevant at this time?	Boyd & le Roux (2017) The happiness that the children gain from participating in the THR activity in turn brings about happiness in their parents. Malcolm et al., 2017 "defy (even if only to a limited degree) the'triad of impairment'"
What would you change and any reasons why can't it be changed	Eg I want to ride more than once per week – can't be changed due to financial restrictions/work/commitments	Boyd & le Roux (2017) – parents mentioned ride more frequently Kendall et al., 2013 – expensive to run – similar outcomes can come from more cost effective

		therapy (Not a study on Autistic Adults – links with next step mentioned in Malcolm et al., 2017 about at least as effective as other therapeutic models.

Practitioner

Can you start with telling be about your role and how you might structure riding sessions for Autistic adults	Understand the role of the practitioner and what they "do"	
Do you see any immediate differences in Autistic Adults during their horse riding sessions?	Immediate benefits between start and end of the session – encourage a narrative answer such as examples within sessions	Boyd & le Roux (2017) – continuing happiness once on the horse & focus
Why do you think this may be?	Expand on what may be done to explain these impacts – tools and activities used	
Do you see any long term effects after their sessions?	Effects noticed outside of sessions and throughout sessions (e.g. if practitioner sees client for other activity based sessions, or notices changes across the sessions such as between session one and session 10)	Boyd & le Roux (2017) The happiness that the children gain from participating in the THR activity in turn brings about happiness in their parents. Malcolm et al., 2017 "defy (even if only to a limited degree) the 'triad of impairment'" & "it builds up over time"
Can you describe any benefits you feel Autistic adults may experience as a result of accessing THR		
Can you identify and describe any barriers faced by Autistic adults who access THR or would like to access THR?		Boyd & le Roux (2017) – parents mentioned ride more frequently Kendall et al., 2013 – expensive to run – similar outcomes can come from more cost effective therapy (Not a study on Autistic Adults – links with next step mentioned in Malcolm et al., 2017 about at least as effective as other therapeutic models.
How could access be improved?		
Do you have any further comments?		
